# Zinc‐Organometallic Framework Vaccine Controlled‐Release Zn^2+^ Regulates Tumor Extracellular Matrix Degradation Potentiate Efficacy of Immunotherapy

**DOI:** 10.1002/advs.202302967

**Published:** 2023-07-13

**Authors:** Lin Ding, Minli Liang, Yuanyuan Li, Mei Zeng, Meiting Liu, Wei Ma, Fuming Chen, Chenchen Li, Rui L. Reis, Fu‐Rong Li, Yanli Wang

**Affiliations:** ^1^ The First Affiliated Hospital (Shenzhen People's Hospital) Southern University of Science and Technology Shenzhen 518055 China; ^2^ Translational Medicine Collaborative Innovation Center The First Affiliated Hospital (Shenzhen People's Hospital) Southern University of Science and Technology Shenzhen 518020 China; ^3^ Guangdong Engineering Technology Research Center of Stem Cell and Cell Therapy Shenzhen Key Laboratory of Stem Cell Research and Clinical Transformation Shenzhen Immune Cell Therapy Public Service Platform Shenzhen 518020 China; ^4^ Clinical Laboratory, Jiaozuo Women's and Child's Hospital Jiaozuo 454001 China; ^5^ School of Pharmacy Guangdong Medical University Dongguan 523109 China; ^6^ Key Laboratory of Tropical Translational Medicine of Ministry of Education School of Pharmacy and The First Affiliated Hospital Hainan Medical University Haikou 570228 China; ^7^ 3B's Research Group I3Bs‐Research Institute on Biomaterials Biodegradables and Biomimetics University of Minho Guimarães 4805–017 Portugal

**Keywords:** immunotherapy, tumor extracellular matrix, vaccine, zinc‐organometallic framework, Zn^2+^

## Abstract

Tumor extracellular matrix (ECM) not only forms a physical barrier for T cells infiltration, but also regulates multiple immunosuppressive pathways, which is an important reason for immunotherapy failure. The cyclic guanosine monophosphate‐adenosine monophosphate synthase‐stimulator of interferon genes (cGAS‐STING) pathway plays a key role in activating CD8^+^ T cells, maintaining CD8^+^ T cells stemness and enhancing the antitumor effect. Herein, a zinc‐organometallic framework vaccine (ZPM@OVA‐CpG) prepared by self‐assembly, which achieves site‐directed release of Zn^2+^ in dendritic cell (DC) lysosomes and tumor microenvironment under acidic conditions, is reported. The vaccine actively targets DC, significantly enhances cGAS‐STING signal, promotes DC maturation and antigen cross‐presentation, and induces strong activation of CD8^+^ T cells. Meanwhile, the vaccine reaches the tumor site, releasing Zn^2+^, significantly up‐regulates the activity of matrix metalloproteinase‐2, degrades various collagen components of tumor ECM, effectively alleviates immune suppression, and significantly enhances the tumor infiltration and killing of CD8^+^ T cells. ZPM@OVA‐CpG vaccine not only solves the problem of low antigen delivery efficiency and weak CD8^+^ T cells activation ability, but also achieves the degradation of tumor ECM via the vaccine for the first time, providing a promising therapeutic platform for the development of efficient novel tumor vaccines.

## Graphical abstract

A novel zinc–organometallic framework vaccine, which releases Zn^2+^ in the dendritic cell (DC) lysosome and tumor microenvironment, amplifies the cyclic guanosine monophosphate‐adenosine monophosphate synthase‐stimulator of interferon genes signal, promotes DC maturation and antigen cross‐presentation, induces strong CD8^+^ T cell‐specific proliferation and activation. In addition, the vaccine up‐regulates the enzyme activity of matrix metalloproteinase–2, degrades various collagen components of tumor extracellular matrix, alleviates the immunosuppressive environment, thus comprehensively enhances immunotherapy.

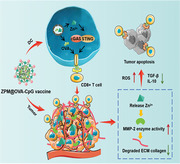

## 1 Introduction

Tumor vaccine could induce specific cellular immunity and humoral immune response, inhibiting the growth, metastasis, and recurrence of tumor cells, is considered one of the most effective means to eradicate tumors.^[^ ^1^ ^]^ However, the antitumor effect of current tumor vaccines is not satisfactory, mainly for the following reasons. First, due to the lack of effective targeted delivery systems, dendritic cells (DCs) are inefficient at capturing antigens, which affects antigen presentation and immune response activation.^[^ ^2^ ^]^ Second, the antigens taken by DCs enter the lysosome and presented to activate CD4^+^ T cells mainly through the major histocompatibility complex (MHC) II pathway, but cannot directly and effectively activate CD8^+^ T cells, which is also an important reason for limiting the tumor‐killing ability of tumor vaccine.^[^ ^3^ ^]^ In addition, there are increasing evidences that tumor extracellular matrix (ECM) is closely related to immunosuppression. Tumor ECM is composed of collagen, hyaluronic acid, elastin, fibronectin, polysaccharide, etc., which combines with tumor cells to form a complex extra‐tumor structure.^[^ ^4^ ^]^ Collagen, the main component of ECM, is the most important source of hardness increase and dense structure formation of tumor ECM.^[^ ^5^ ^]^ Studies had shown that, increased tumor ECM hardness inhibited the ability of DCs to recognize and internalize tumor antigens, leading to failure of T cells activation.^[^ ^6^ ^]^ The dense structure of tumor ECM formed a physical barrier, blocking the deep penetration of antibodies and T cells, resulting in a decrease in the killing ability of T cells.^[^ ^7^ ^]^ High‐density ECM collagen guided macrophages to obtain immunosuppressive phenotype, leading to T cell dysfunction.^[^ ^8^ ^]^ ECM barrier seriously hinders the tumor‐killing function of CD 8^+^ T cells, which is a crucial reason for the failure of immunotherapy. How to effectively break down the immunosuppressive environment formed by ECM barrier is an urgent problem to be solved. To sum up, developing novel tumor vaccine to solve such problems as low efficiency of antigen targeted delivery, insufficient induction of CD8^+^ T cells and difficulty in breaking through tumor ECM barrier are the core issues in vaccine development.

In recent years, researchers had found that zinc ion (Zn^2+^) played an important role in regulating immune response and tumor therapy.^[^ ^9^ ^]^ Reports pointed out that Zn^2+^ was involved in the regulation of toll‐like receptor 4‐mediated signaling pathway, promoting monocytes to secrete tumor necrosis factor‐*α* (TNF‐*α*), interleukin‐1*β*, interferon‐*γ* (IFN‐*γ*) and other inflammatory factors.^[^ ^10^ ^]^ Zn^2+^ up‐regulated the expression of costimulatory molecules of CD80 and CD86, thus promoting the maturation and antigen presentation of DCs.^[^ ^11^ ^]^ Zn^2+^ was an essential substance for interleukin‐2 (IL‐2) to stimulate T cells activation, and zinc deficiency would seriously destroy T lymphocyte mediated cellular immune response.^[^ ^12^ ^]^ Science article proved that free Zn^2+^ could enhance the activity of cyclic guanosine monophosphate‐adenosine monophosphate synthase (cGAS) enzyme and assist the activation of cGAS‐stimulator of interferon genes (cGAS‐STING) pathway by promoting the separation of cGAS‐DNA phase.^[^ ^13^ ^]^ The cGAS‐STING signaling pathway is an important innate immune pathway that plays a crucial role in cancer immune surveillance.^[^ ^14^ ^]^ STING activation has been shown to promote the cancer immune cycle and transform the immunosuppressive tumor microenvironment into an immune‐supported microenvironment.^[^ ^15^ ^]^ cGAS‐STING activation can maintain the stemness of CD8^+^ T cells and enhance the anti‐tumor effect.^[^ ^16^ ^]^ Therefore, Zn^2+^ has significant potential in assisting immune activation.

In addition, Zn^2+^ participates in the regulation of ECM‐degrading enzyme activity. Matrix metalloproteinases (MMPs) are a family of Zn^2+^ dependent endogenous proteolytic enzymes, whose main function is to degrade ECM and basement membrane. Studies pointed out that Zn^2+^ participation was a necessary condition for the activation of MMPs.^[^ ^17^ ^]^ Therefore, Zn^2+^ has the ability to regulate ECM degradation.

Moreover, our previous research suggested that zinc‐based nanomaterials accumulation could induce tumor‐reactive oxygen species (ROS) production.^[^ ^18^ ^]^ Elevated ROS levels can initiate apoptosis and inducing high levels of ROS in tumor is one of the important means of tumor treatment.^[^ ^19^ ^]^ However, the high content of glutathione (GSH) in the tumor microenvironment can eliminate excessive ROS in the tumor and reduce the damage of ROS to cancer cells. It is found that Zn^2+^ could chelate with GSH, thus reducing GSH content.^[^ ^20^ ^]^ In addition, malignant tumors such as liver, gallbladder, prostate, lung, cervical, and myeloma detected a decrease in Zn^2+^ concentration in the tissue, which indicated insufficient zinc content in the tumor tissue.^[^ ^21^ ^]^ Therefore, increasing Zn^2+^ delivery in tumor tissue can help reduce GSH level, increase ROS accumulation in tumor, and initiate tumor apoptosis.

**Figure 1:**
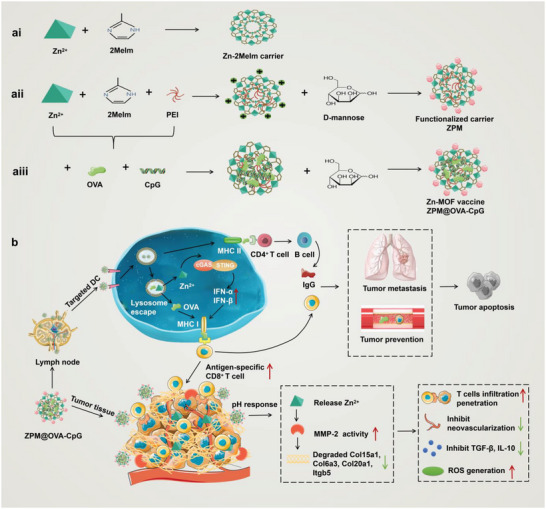
Preparation process and enhance immunotherapy mechanisms of ZPM@OVA‐CpG vaccine.

In conclusion, Zn^2+^ not only plays a key role in regulating immune activation, but also has a potentially critical function in degrading the ECM barrier and initiating tumor apoptosis. Herein, we constructed a novel zinc‐organometallic framework vaccine (Zn‐MOF vaccine/ZPM@OVA‐CpG) with controlled release of Zn^2+^ in pH response based on coordination self‐assembly and targeted ligand modification techniques (**Figure** **1**a). On the one hand, the vaccine actively targeted DCs to improve the efficiency of antigen uptake and presentation. The vaccine released Zn^2+^ and polyethyleneimine (PEI) in DC lysosome, induced lysosome swelling, promoted antigen cross‐presentation, and regulated CD8^+^ T cells activation. Moreover, the released Zn^2+^ activated the cGAS‐STING pathway and maintained strong CD8^+^ T cells immunity. On the other hand, the vaccine reached the tumor site, releasing Zn^2+^ in acidic tumor microenvironment, improving the activity of MMP‐2, promoting ECM collagen degradation, and enhancing tumor invasion and killing of CD8^+^ T cells (Figure 1b). The Zn‐MOF vaccine is a novel vaccine with three functions of antigen targeted delivery, immune system activation, and degradation of tumor ECM barrier. It is expected to solve the bottleneck of insufficient vaccine efficacy and provide a promising new vaccine technology for immunotherapy.

## 2 Results and Discussion

### 2.1 Zn^2+^ Stimulates Cgas‐Sting Pathway Activation, Up‐Regulates Mmp‐2 Activity, and Induces Ros Production In Tumor Cells

Activated of cGAS‐STING pathway could stimulate immune cells to secrete of interferon‐*α* (IFN‐*α*) and interferon‐*β* (IFN‐*β*).^[^ ^22^ ^]^ We compared the activation ability of different metal ions (Mn^2+^, Zn^2+^, Cu^2+^, K^+^, Fe^2+^, Ca^2+^, Mg^2+^) at three concentrations of 250, 500, 1000 µM on the cGAS‐STING pathway, and the results showed that Zn^2+^ had the strongest ability to stimulate DCs to secrete IFN‐*α* and IFN‐*β* (Figure S1a, Supporting Information).

Then we examined the effect of Zn^2+^ on the activity of MMPs. Mouse fibroblasts (l929 cells) are the main cells secreting MMPs. We cocultured 1000 µM of Zn^2+^ with l929 cells for 24 h to detect the activity of MMP‐2/9. The results showed that the enzyme active bands of MMP‐9 did not change significantly, while the bands of MMP‐2 were thickened significantly (Figure S1b, Supporting Information). MMP‐2 is a gelatin‐like enzyme involved in the degradation of collagen in ECM. The non‐catalytic C‐terminal fragment of MMP‐2 gene has anti‐angiogenesis and anti‐tumor properties.^[^ ^23^ ^]^ These results indicate that Zn^2+^ has the potential to degrade ECM collagen by up‐regulating MMP‐2 activity.

Subsequently, we examined ROS production induced by Zn^2+^ in tumor cells. Zn^2+^ (1000 µM) was co‐cultured with mouse melanoma cells (B16‐OVA cells) for 6 h, and the intracellular ROS production was detected, as shown in Figure S1c, Supporting Information. Compared with control, obvious ROS production (green fluorescence) was observed in B16‐OVA cells, and the number of cells decreased, indicating that ROS production induced tumor apoptosis. We used CCK‐8 reagent to detect the cytotoxicity of Zn^2+^ at different concentrations (0, 250, 500, 1000, 1500 µM) to DCs, T cells, and B16‐OVA cells respectively, as shown in Figure S1d, Supporting Information. Zn^2+^ at 1000 µM concentration showed obvious toxicity to B16‐OVA tumor cells.

The above results indicated that, Zn^2+^ significantly activated the cGAS‐STING pathway, effectively up‐regulated the MMP‐2 activity and induced tumor apoptosis by increasing the accumulation of Zn^2+^ in tumor cells.

### 2.2 Preparation and Characterization of Zn‐2MeIm Carrier, ZPM Carrier, and ZPM@OVA‐CpG Vaccine

**Figure 2:**
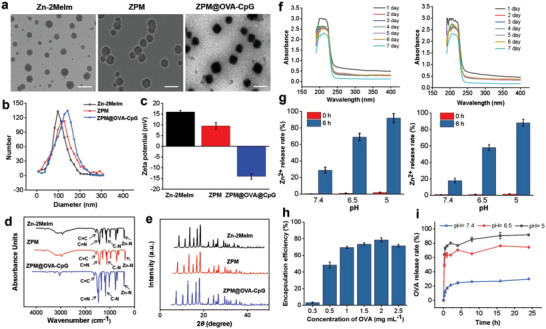
Characterization of physicochemical properties of Zn‐2MeIm, ZPM, and ZnPM@OVA‐CpG. a) Morphological observation by TEM, scale is 200 nm; b) particle size distribution; (c) zeta potential, data presented as mean ± SD (*n* = 3); d) FTIR spectrum; e) PXRD peaks; f) UV absorption peaks change, left is ZPM, right is ZPM@OVA‐CpG; g) statistics of the Zn^2+^ release rate in different pH, left is ZPM, right is ZPM@OVA‐CpG, data presented as mean ± SD (*n* = 3); h) encapsulation efficiency of ZPM@OVA‐CpG vaccine on OVA, data presented as mean ± SD (*n* = 3); i) OVA release efficiency at different pH of ZPM@OVA‐CpG vaccine, data presented as mean ± SD (*n* = 3).

Zn‐2MeIm carrier is a zinc‐organometallic frame material formed by the coordination of Zn^2+^ and 2‐methylimidazole (2MeIm) in aqueous solution (Figure 1ai). ZPM carrier was synthesized by mixing Zn^2+^, PEI, and 2MeIm in aqueous solution, and then D‐mannose (Man) was coated on the carrier surface (Figure 1aii). ZPM carry ovalbumin (OVA) and adjuvants (CpG) to form Zn‐MOF vaccines (ZPM@OVA‐CpG) (Figure 1aiii). The reason for selecting PEI modification is that PEI has protonation effect, which can promote the osmotic swelling of lysosome, and thus facilitate the lysosome escape of antigen.^[^ ^24^ ^]^ Man is modified because a large number of Man‐receptors are expressed on the surface of DCs, and therefore the carrier could actively targeted DCs.^[^ ^25^ ^]^ After modification by PEI and Man, the ZPM@OVA‐CpG could actively target DCs to deliver antigens and activate CD8^+^ T cells through antigens lysosome escape. From the transmission electron microscopy (TEM) images (**Figure** **2**a) and scanning electron microscope (SEM) images (Figure S2, Supporting Information), can be seen that Zn‐2MeIm carrier and ZPM carrier displayed polyhedral structure, while ZPM@OVA‐CpG presented approximate spherical particles due to the coating of OVA and CpG. Dynamic light scattering indicated a hydrodynamic size of 98 nm, 128 nm, and 142 nm for Zn‐2MeIm, ZPM, and ZPM@OVA‐CpG, respectively (Figure 2b). Zeta potential of 15.95 ± 0.78 mV, 9.48 ± 1.56 mV, and −14.04 ± 1.26 mV for Zn‐2MeIm, ZPM, and ZPM@OVA‐CpG, respectively (Figure 2c). Zn‐2MeIm was positively charged due to the presence of unsaturated Zn^2+^ coordination sites on the surface.^[^ ^26^ ^]^ The zeta potential of the functionalized ZPM carrier decreased because the surface was coated with negative Man. The negative zeta potential of ZPM@OVA‐CpG was due to loading the large amount of negatively charged OVA and CpG. The size and zeta potential changes indicated the effectiveness encapsulation of antigens. The C≐C peaks at 1624 cm^−1^, C≐N peaks at 1471 cm^−1^, C–N peaks at 1228 cm^−1^ and Zn–N peaks at 486 cm^−1^ can be observed in the FTIR spectrum, indicating the generation of self‐assembled structures coordinated by Zn^2+^ and 2MeIm (Figure 2d). Powder X‐ray diffraction (PXRD) pattern of Zn‐2MeIm, ZPM, and ZPM@OVA‐CpG were consistent, indicating that the crystal structure of Zn‐2MeIm was not affected by PEI, Man, and OVA‐CpG (Figure 2e).

ZPM@OVA‐CpG appeared as white powder after drying (Figure S3a, Supporting Information) and were well dispersed in PBS (Figure S3b, Supporting Information). We dispersed ZPM and ZPM@OVA‐CpG in PBS and monitored their UV absorption peaks for 7 days. The UV absorption peaks of ZPM and ZPM@OVA‐CpG were 210 nm and 219 nm, respectively, and the peaks were relatively stable after 6 days of storage in PBS, and slightly decreased on the 7th day, showing good stability (Figure 2f). We also visually observed the acid response cleavage effect of the carrier and vaccine. ZPM and ZPM@OVA‐CpG were placed in PBS with different pH environments (pH 7.4 mimics normal extracellular fluid environment, pH 6.5 mimics tumor acid environment and pH 5 mimics lysosome acidity condition), the solutions showed white turbidized state at 0 h. After 6 h, the solutions became clear under the conditions of pH 6.5 and 5, while the solutions remained turbidized under the conditions of pH 7.4 (Figure S3c, Supporting Information). We measured the amount of dissolved Zn^2+^ in the supernatant. Both the ZPM and the ZPM@OVA‐CpG showed similar characteristics of releasing Zn^2+^ in pH response. Within 6 h, the release rate of Zn^2+^ reached ≈60% and 85% at pH 6.5 and 5, respectively (Figure 2g). For further verification, morphology changes of ZPM@OVA‐CpG after different pH treatment for 6 h were observed by SEM. As shown in Figure S3d, Supporting Information, ZPM@OVA‐CpG maintained a polyhedral structure at pH 7.4, while at pH 6.5 for 6 h, the carrier had been partially degraded, while at pH 5 for 6 h, most of the carriers had been degraded and lost the original shape. The results directly demonstrated the acid response degradation characteristics of ZPM@OVA‐CpG.

To test the loading efficiency of the ZPM on OVA, different concentrations of OVA were put into the preparation process. As shown in Figure 2h, with the increase of OVA concentration, the encapsulation efficiency of OVA gradually increased. When the concentration of OVA reached 2 mg mL^−1^, the encapsulation efficiency of OVA reached the highest of 78.5%, and then began to decline. The results showed that the ZPM carrier had high loading efficiency for OVA. To investigate the efficiency of ZPM@OVA‐CpG to release OVA, ZPM@OVA‐CpG were dispersed in PBS with pH of 5, 6.5, and 7.4. As shown in Figure 2i, in the pH 7.4 environment, the maximum release rate of OVA was only 29% within 24 h, protecting antigen nonspecific degradation. With the decrease of solution pH, the OVA release rate increased, under the acidic condition of pH 6.5, the release amount of OVA reached 74%. The highest release rate of OVA reached ≈92% under the pH 5. These results indicated that the vaccine was stable in normal physiological environment, protected the non‐specific degradation of the antigen, and at the same time, the vaccine had a sensitive acid‐reactive cleavage effect to ensure the targeted release of the antigen in the lysosomes.

### 2.3 Biosafety Analysis

**Figure 3:**
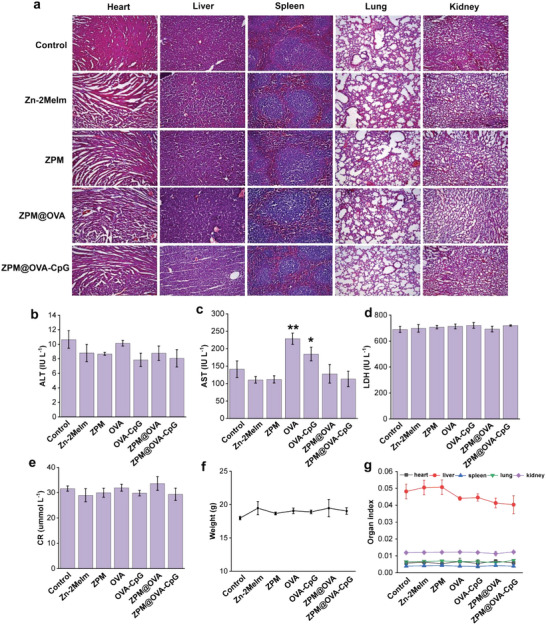
Biosafety analysis. a) Pathological section observation; b‐e) blood biochemical index detection, (b) ALT, (c) AST, (d) LDH, (e) CR; f) mice weight; g) organ index statistics. *p < 0.05; **p < 0.01, compared of control. Data presented as mean ± SD (*n* = 5).

The biosafety of materials is the primary concern. Therefore, the key indicators such as the weight, organ index, blood biochemical indicators, organ pathological changes were evaluated to assess the biocompatibility of ZPM@OVA‐CpG. Female C57BL/6 mice (5–6 weeks of age) were subcutaneously injected in different experimental groups three times, the interval between injections was 7 days. After three injections, mice were weighed, blood collected, and dissected for biosafety evaluation. From the pathological sections of the main organs, no obvious pathological changes were observed after injecting each experimental group (**Figure** **3**a and Figure S4a, Supporting Information). In addition, we detected the changes in several common indicators reflecting liver, kidney function, myocardial injury, and inflammatory reaction, including alanine aminotransferase (ALT), aspartate aminotransferase (AST), lactic dehydrogenase (LDH) and creatinine (CR). As shown in Figure 3b‐e, there were no significant changes in ALT, CR, and LDH indexs, indicating that each experimental group would not cause liver, kidney, and inflammatory reaction. The normal reference range of blood biochemical indicators: ALT (13.3–72.3 IU L^−1^), AST (35.7–135.7 IU L^−1^), CR (28–37 µmol L^−1^), LDH (471–717 IU L^−1^).^[^ ^27^ ^]^ The OVA and OVA‐CpG groups caused the elevation of AST, but the elevation of a single index could not be clinically determined as tissue injury. The body weight (Figure 3f) and organ index (Figure 3g) of mice did not change significantly. The above results showed that the ZPM@OVA‐CpG vaccine has good biocompatibility and biosafety.

In addition, we characterized the cytotoxicity of each material group to DCs and T cells. We successfully isolated and induced DCs and T cells from PBMC by previous methods.^[^ ^28^ ^]^ As shown in Figure S4b, Supporting Information, monocytes were isolated from PBMC on day 1, cells began to grow in clusters, and dendritic branches began to appear around the cells on day 4, by day 7, the DCs grew in clusters more obviously, showing a semi suspended state, dendritic branches were obviously seen around the cells (as shown by the red arrows), which indicating that DCs were successfully induced and cultured in vitro.^[^ ^29^ ^]^ T cells were collected from the PBMC supernatant, and IL‐2 was added for induction and culture. As shown in Figure S4c, Supporting Information, T cells were smaller than DCs and grew in a circular single‐cell suspension state. We cocultured of Zn‐2MeIm, ZPM, and ZPM@OVA‐CpG with DCs and T cells at different concentrations for 24 h, and found that even if the concentration reached 100 mg L^−1^, there were no obvious toxicity to DCs and T cells (Figure S4d,e, Supporting Information), which further evidence of low cytotoxicity.

### 2.4 ZPM@OVA‐CpG Vaccine Target DCs to Promote Antigen Uptake and Presentation

**Figure 4:**
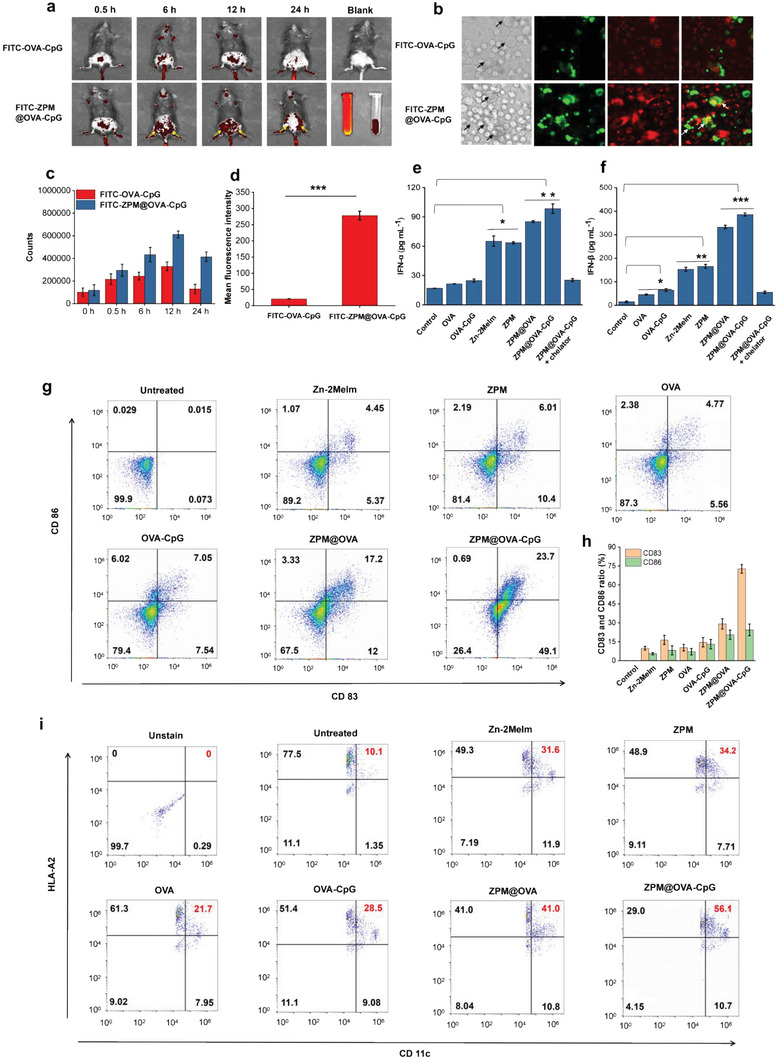
ZPM@OVA‐CpG vaccine target DCs to promote antigen uptake and presentation. a) In vivo imagings show the distribution of OVA in mice (yellow arrows indicate the inguinal lymph nodes), left tube is FITC‐OVA‐CpG, right tube is FITC‐ZPM@OVA‐CpG; b) confocal microscope imagings showing DCs uptake and lysosome escape of OVA (black arrows indicate DCs, white arrows indicate the escaped OVA); c) fluorescence intensity statistics of OVA at different time in mice, data presented as mean ± SD (*n* = 3); d) average fluorescence intensity statistics of OVA in DCs data presented as mean ± SD (*n* = 3); e) IFN‐*α* content released by DCs, data presented as mean ± SD (*n* = 6); f) IFN‐*β* content released by DCs, data presented as mean ± SD (*n* = 6); g) detection of CD83 and CD86 markers of DC maturation by flow cytometer; h) CD 83 and CD86 ratio of DCs after different treatment, data presented as mean ± SD (*n* = 3); i) detection of MHC I complex of HLA‐A2 by flow cytometer. *p < 0.05; **p < 0.01; ***p < 0.001.

Lymph node is one of the most important immune organs. It is the main place for receiving antigen stimulation to generate immune response. It gathers a large number of DCs, and these cells are adjacent to the initial T cells, which can achieve rapid presentation after antigen ingestion.^[^ ^30^ ^]^ Therefore, effective delivery of tumor vaccine to lymph nodes is one of the important means to improve vaccine efficiency. We injected fluorescein isothiocyanate labeled OVA‐CpG (FITC‐OVA‐CpG) and FITC‐ZPM@OVA‐CpG into mice subcutaneously through the tail, and observed the distribution of antigen. As shown in **Figure** **4**a, after 0.5 h of injection, the antigen began to appear in the abdomen of mice, within 6 to 12 h, the FITC‐ZPM@OVA‐CpG group in mice was significantly higher than that of FITC‐OVA‐CpG group, and mainly concentrated in leg inguinal lymph nodes. Mouse inguinal lymph nodes were distributed bilaterally at the base of the thigh (as shown by the yellow arrows), while FITC‐OVA‐CpG group did not observe lymph node aggregation.^[^ ^31^ ^]^ After 24 h, FITC‐OVA‐CpG group was almost metabolized, while FITC‐ZPM@OVA‐CpG group was still significantly aggregated in mice inguinal lymph nodes. 24 h after injection, the inguinal lymph nodes of mice were removed and their fluorescence signals were observed, as shown in Figure S5, Supporting Information, there were no fluorescence signals in the blank control group and FITC‐OVA‐CpG group, while the fluorescence signals in the lymph nodes of FITC‐ZPM‐OVA group were strong, indicating that ZPM‐OVA‐CPG effectively targeted lymph nodes. Fluorescence intensity signal showed that the antigen of FITC‐ZPM@OVA‐CpG group was 2–3 times compared to that of OVA‐CpG in mice (Figure 4c). The above results indicated that, ZPM@OVA‐CpG vaccine effectively delivered more antigens to lymph nodes and prolonged the residence time of antigens in vivo.

After the antigen is delivered to the lymph node, the efficiency of antigen uptake and presentation by DCs will affect the subsequent immune response. The ZPM carrier was modified by Man, and Man‐receptor were expressed in large quantities on the surface of DCs.^[^ ^25^ ^]^ And dependent on Man‐receptor mediated, improving the antigen uptake ability of DCs has been demonstrated.^[^ ^32^ ^]^ Therefore, ZPM@OVA‐CpG could actively target DCs and were taken up by DCs through receptor‐mediated pathways. As shown in Figure 4b, DCs (black arrows) had very low uptake of FITC‐OVA‐CpG, while DCs uptake of FITC‐ZPM@OVA‐CpG had greatly increased and most of the green antigen fluorescence did not overlap with the red lysosome fluorescence (white arrows), indicating that antigen to achieve lysosome escape. The uptake of FITC‐ZPM@OVA‐CpG by DC clusters were more significant. As shown in Figure S6, Supporting Information, the whole cell cluster showed a green antigen signal. Mean fluorescence intensity of FITC‐ZPM@OVA‐CpG in DCs was 13 times that of FITC‐OVA‐CpG (Figure 4d). These results indicated the effective targeting of FITC‐ZPM@OVA‐CpG to DCs, and greatly increased the uptake of antigen by DCs.

We then examined the stimulation of cGAS‐STING activation in each experimental group. As shown in Figure 4e,f, Zn‐contained groups (Zn‐2MeIm, ZPM, ZPM@OVA, and ZPM@OVA‐CpG) effectively stimulated IFN‐*α* and IFN‐*β* secretion, the effect were significantly better than that of zinc free groups (OVA and OVA‐CpG). ZPM@OVA‐CpG group had the most significant effect in stimulating cGAS‐STING pathway. However, after ZPM@OVA‐CpG was added with Zn^2+^ chelator, the stimulated secretion of IFN‐*α* and IFN‐*β* were significantly reduced, further demonstrating the regulatory effect of Zn^2+^ on the activation of cGAS‐STING pathway.

Mature DCs highly express MHC molecules, costimulatory molecules, and adhesion molecules, which can effectively activate initial T cells and are in the central link of starting, regulating, and maintaining immune response.^[^ ^33^ ^]^ Therefore, the maturity of DCs is very important for activating subsequent immune response. CD83 is involved in antigen presentation and lymphocyte activation, which is a specific marker of DCs maturation.^[^ ^34^ ^]^ CD86 binds to the receptor CD28 protein on the surface of T cells, giving signals of initial T cells activation, and promoting T cells activation, proliferation, and differentiation.^[^ ^35^ ^]^ Immature DCs low express of CD83 and CD86, and the increased expression of the two proteins can verify the maturity of DCs. As shown in Figure 4g, the expression of CD83 and CD86 were increased in all treatment groups. The proportion of CD11c^+^ CD83^+^ was 72.8%, CD11c^+^CD86^+^ was 24.39% in ZPM@OVA‐CpG group (Figure 4h). This result was significantly higher than the activation ability of OVA or OVA‐CpG on DCs, indicating that the ZPM@OVA‐CpG has the strongest ability to stimulate DCs maturation.

After DCs ingest the antigen, they present the antigen information to T cells through MHC molecules, thus activating the specific immune response of T cells. Mature DCs usually express MHC class II molecules on their surfaces, and the antigens are directed to MHC class II molecules by the lysosomal pathway and presented to activate CD4^+^ T cells.^[^ ^36^ ^]^ CD8^+^ T cells are key immune cells involved in killing tumor cells, however, their activation is mainly dependent on antigen binding presented by MHC I complex.^[^ ^37^ ^]^ In the above studies, we have observed that ZPM@OVA‐CpG promoted the antigens lysosomes escape, and the efficiency of antigen cross‐presentation can be determined by detecting the expression of MHC I complex on the DCs surface. The human MHC is called human leucocyte antigen (HLA). The most common subtype of MHC I in the Chinese population is HLA‐A2.^[^ ^38^ ^]^ As shown in Figure 4i, the expression of HLA‐A2 was increased in each group after treatment, the proportion of HLA‐A2 in Zn‐2MeIm and ZPM groups were 31.6% and 34.2%, respectively, which were better than that of OVA (21.7%) and OVA‐CpG (28.5%) groups. The proportion of HLA‐A2 in ZPM@OVA‐CpG group was 56.1%, which was the highest among all groups and the efficiency of antigen cross‐presentation was increased 46% compared untreated group. The results showed that the ZPM@OVA‐CpG group significantly promoted antigen cross‐presentation.

### 2.5 ZPM@OVA‐CpG Vaccine Induces CD8^+^ T cells proliferation, activation and tumor killing

**Figure 5:**
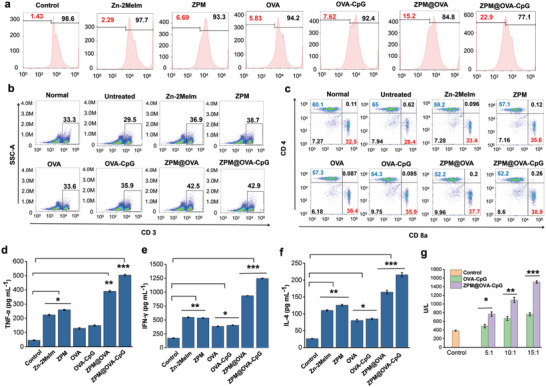
CD8^+^ T cells proliferation activation and tumor killing. a) Flow cytometry determined the proliferation rate of CFSE‐labeled T cells; b) flow cytometry detected the total number of T cells in spleen; c) flow cytometry detected the CD8^+^ T cells and CD4^+^ T cells ratio in spleen; d) TNF‐*α* content released by CD8^+^ T cells, data presented as mean ± SD (*n* = 6); e) IFN‐*γ* content released by CD8^+^ T cells, data presented as mean ± SD (*n* = 6); f) IL‐4 content released by CD8^+^ T cells, data presented as mean ± SD (*n* = 6); g) release of lactate dehydrogenase from T cells and B16 cells co‐cultured in different proportions, (*T* cells: B16‐OVA cells ratio of 5:1, 10:1, 15:1, respectively), data presented as mean ± SD (*n* = 6). *p < 0.05; **p < 0.01; ***p < 0.001.

T cells activation and proliferation produce effector T cells that trigger tumor‐specific killing.^[^ ^39^ ^]^ Therefore, we examined the effect of each experimental group on T cells proliferation. We extracted T cells from PBMC, co‐cultured with induced DCs by each experimental group, and monitored the proliferation of T cells through the 5(6) ‐carboxyfluorescein diacetate succinimide (CFSE) method. T cells proliferation causes CFSE signaling to left shift. As shown in **Figure** **5**a, T cells in control group was almost not increased. The efficiency of T cells proliferation in Zn‐2MeIm, ZPM, OVA, OVA‐CpG, ZPM@OVA, and ZPM@OVA‐CpG group were 2. 29%, 6.69%, 5.83%, 7.62%, 15.2%, and 22.9%, respectively. ZPM@OVA‐CpG significantly improved the proliferation capacity of T cells in vitro. Subsequently, we examined the ability of each group to induce T cell proliferation in vivo. The spleen is the largest immune organ and the place where immune cells are stimulated by antigen to proliferate and differentiate.^[^ ^40^ ^]^ A large number of T lymphocytes are produced in the spleen and directly participate in cellular immunity. The spleens of mice treated with each group were collected and OVA‐specific T cells were isolated using T‐select H‐2Kb OVA Tetramer SIINFEKL‐PE. The total T cells were labeled with CD3 antibody and detected by flow cytometry. The results were shown in Figure 5b that the proportion of T cells in the spleen of untreated tumor mice was the lowest (29.5%). ZPM@OVA‐CpG groups (42.9%) increased the total number of spleen T cells by 13.4% compared to untreated tumor mice and 9.6% compared normal group (the healthy mice were treated without any treatment), which would greatly improve the efficiency of an immune response. These results indicated the ZPM@OVA‐CpG vaccine showed significant promotion of T cells proliferation both in vitro and in vivo.

We further explored the generation of CD4^+^ and CD8^+^ T cells in spleen (Figure 5c). We found that the proportion of CD8^+^ T cells in untreated tumor mice was only 26.4%, the CD4^+^/CD8^+^ T cells ratio was 2.46, which exceeded the normal range of 1.4‐2, indicating the disorder of the immune system.^[^ ^41^ ^]^ While the CD4^+^/CD8^+^ T cells ratio of each experimental group was within the normal range after treatment, indicating the effectiveness of treatment. The ZPM@OVA‐CpG vaccine group had the highest percentage of CD8^+^ T cells (38.9%), which was 12.5% higher than untreated tumor mice. These results indicated the ZPM@OVA‐CpG vaccine induced a strong proliferation of CD8^+^ T cells.

Cytokines play an important role in immune regulation. There are different subtypes of CD8^+^ T cells. Tc1 type CD8^+^ T cells can release Th1 cytokines such as IFN‐*γ* or TNF‐*α*, participate in the regulation of cellular immunity, assist cytotoxic T cell differentiation, and mediate cellular immune response. Tc2 type CD8^+^ T cells regulate effector immunity and adjuvant immune response by secreting Th2 cytokine such as interleukin‐4 (IL‐4).^[^ ^42^ ^]^ IFN‐*γ* and TNF‐*α* are two important pro‐inflammatory cytokines, which can promote the activation of macrophages, upregulate the expression of antigen processing and presentation molecules, promote the growth and activation of Th1 cells, enhance the function of NK cells, regulate the function of B cells, and kill tumor cells by inducing apoptosis.^[^ ^42^ ^]^ IL‐4 is a multipotent cytokine mainly produced by activated T lymphocytes, mast cells, and basophils, which can promote T cell division and stimulate B cells to produce immunoglobulin, with an anti‐tumor effect.^[^ ^43^ ^]^ Therefore, we examined the secretion of cytokines by CD8^+^ T cells to assess whether CD8+T cells were effectively activated. As shown in Figure 5d‐f, Zn‐2MeIm, and ZPM groups showed better performance than the OVA and OVA‐CpG groups in stimulating secretion of these cytokines. The secretion of TNF‐*α*, IFN‐*γ*, and IL‐4 in ZPM@OVA‐CpG group was 11 times, 7 times, and 8 times relative untreated group, suggesting the CD8^+^ T cells were activated and secreted large amounts of immune cytokines, inducing a strong immune response.

Finally, we investigated the ability of activated CD8^+^ T cells to kill specific tumor cells. OVA‐CpG and ZPM@OVA‐CpG groups induced DC were cocultured with sorted CD8^+^ T cells for 3 days, then used B16‐OVA as target cells and above CD8^+^ T cells as effector cells cocultured for 24 h at different effect‐target ratios (T cells: B16‐OVA cells ratio of 5:1, 10:1, 15:1, respectively). The apoptosis of tumor cells was detected by a lactate dehydrogenase kit. As shown in Figure 5g, the apoptosis‐inducing ability of the ZPM@OVA‐CpG group was significantly better than that of the OVA‐CpG group. And with the increase of effect target ratio, the apoptosis‐inducing ability was stronger.

### 2.6 ZPM@OVA‐CpG Vaccine Degrades Tumor ECM and Relieves the Immunosuppressive Environment

Tumor ECM forms a physical barrier for T cells infiltration, regulates the tumor immunosuppressive environment, and causes T cells dysfunction, which is a major reason for the failure of immunotherapy.^[^ ^44^ ^]^ It is worth noting that collagen is not only the main component of tumor ECM and the main cause of tumor hardness increase, but also closely related to angiogenesis, tumor cell adhesion, migration, and immunosuppressive environment.^[^ ^45^ ^]^ For example, collagen and fibronectin synthesis promoted the release of tumor‐associated chemokines, which enhanced tumor cell invasion.^[^ ^46^ ^]^ The high density of collagen further promoted the transformation of macrophages into M2 type macrophages. These M2 macrophages further enhanced immunosuppression by secreting more transforming growth factor‐*β* (TGF‐*β*).^[^ ^47^ ^]^ Researchers delivered collagen‐degradable enzymes to promote tumor ECM degradation and relieved the immunosuppressive environment.^[^ ^48^ ^]^ Therefore, targeted degradation of ECM collagen will be an effective method to break the barrier of ECM.

**Figure 6:**
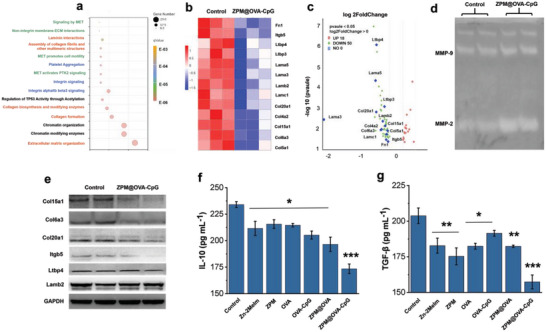
ZPM@OVA‐CpG vaccine degrades tumor ECM mechanism. a) The bubble maps showing the top 15 significant enrichment functions of ZPM@OVA‐CpG vaccine on tumor cells; b) significantly down‐regulated genes heat map; c) volcano map of significant changes in genes distribution; d) enzyme activity bands of MMP‐2/9; e) western blot verification; f) IL‐10 content released by B16‐OVA cells, data presented as mean ± SD (*n* = 6); g) TGF‐*β* content released by B16‐OVA cells, data presented as mean ± SD (*n* = 6). *p < 0.05; **p < 0.01; ***p < 0.001, compared of control.

We used transcriptome sequencing technology to detect the effect of ZPM@OVA‐CpG vaccine on tumor cells, and the results showed that the top 15 functions with the most significant effect of the ZPM@OVA‐CpG vaccine on tumor cells were mostly related to ECM formation and signal transduction (red marks were associated with ECM formation, blue marks were related to angiogenesis, green marks were related to tumor invasion and metastasis signal transduction) (**Figure** **6**a). Heat map results showed that the genes significantly down‐regulated after ZPM@OVA‐CpG vaccine treatment were the main component protein genes of ECM, including transformed growth factor *β*‐binding protein family (Ltbp4, Ltbp3), laminin family (Lama5, Lama3, Lamb2, Lamc1), collagen family (Col20a1, Col4a2, Col15a1, Col6a3, Col5a1), fibritin (Fn1) and integrin protein (Itgb5) (Figure 6b). The volcanic map showed the differential distribution of each significantly down‐regulated genes (Figure 6c). These results indicated ZPM@OVA‐CpG vaccine mainly affect tumor ECM‐related components and signal regulation.

We tested the activity of MMPs, and the results showed that the ZPM@OVA‐CpG vaccine significantly up‐regulated the activity of MMP‐2 (Figure 6d). Western blotting was used to detect the expression of the above representative proteins in tumor cells treated with the ZPM@OVA‐CpG vaccine, and the results showed that the expression levels of Col15a1, Col6a3, Col20a1 and Itgb5 were significantly down‐regulated. The expression of Ltbp4 and Lamb2 did not change significantly (Figure 6e). The average gray value statistics of the strips also showed similar results (Figure S7a‐f and Tables S1,2, Supporting Information). As shown in Figure S7g, Supporting Information, OVA‐CpG and ZPM@OVA‐CpG + chelator groups had no significant effect on Col15a1, Col6a3, Col20a1, and Itbg5. These results further support the effectiveness of ZPM@OVA‐CpG in degrading ECM. It is reported that, Col6a3's C5 endotrophin peptide enhanced cancer cell metastasis by inducing TGF‐*β*‐dependent epithelial‐mesenchymal transition, and Col6a3 endotrophin peptide also promoted tumor angiogenesis by increasing endothelial cell recruitment.^[^ ^49^ ^]^ Some studies had shown that the expression level of Col15a1 and Col20a1 were high in some cancers, including lung cancer, gastric cancer, colorectal cancer, etc.,^[^ ^50^ ^]^ suggesting that Col15a1, Col20a1 may be involved in tumor growth and metastasis.^[^ ^51^ ^]^ Integrins are important ECM receptor proteins on the cell surface. The excess collagen binds to integrins on the cell membrane and mediates the adhesion of tumor cells.^[^ ^52^ ^]^

We then measured the secretion of immunosuppressive cytokines interleukin 10 (IL‐10) and TGF‐*β* by tumor cells in each treatment group, the results showed that the ZPM@OVA‐CpG vaccine group significantly inhibited the secretion of these two cytokines (Figure 6f,g). IL‐10 and TGF‐*β* are immunosuppressors secreted by tumor cells. Studies had shown that they inhibited the activity of T cells and NK cells, promoted the differentiation and expansion of regulatory T cells, and enhanced the body's tolerance to its own tissues and foreign antigens.^[^ ^53^ ^]^ They also inhibited a variety of pro‐inflammatory cytokines produced by immune cells, such as IFN‐*γ*, TNF‐*α*, so as to reduce the intensity of inflammatory response.^[^ ^54^ ^]^

These results suggested that ZPM@OVA‐CpG vaccine degraded various collagen components and integrins of ECM by up‐regulating MMP‐2 activity, thus not only softening tumor ECM, but also inhibiting tumor angiogenesis, tumor cell adhesion, and alleviating the tumor immunosuppressor environment. In addition, ZPM@OVA‐CpG vaccine treatment effectively stimulated ROS production in tumor cells and initiated tumor apoptosis (Figure S8, Supporting Information).

### 2.7 ZPM@OVA‐CpG Vaccine Inhibit Tumor Growth, Prevent Tumor Formation and Metastasis

**Figure 7:**
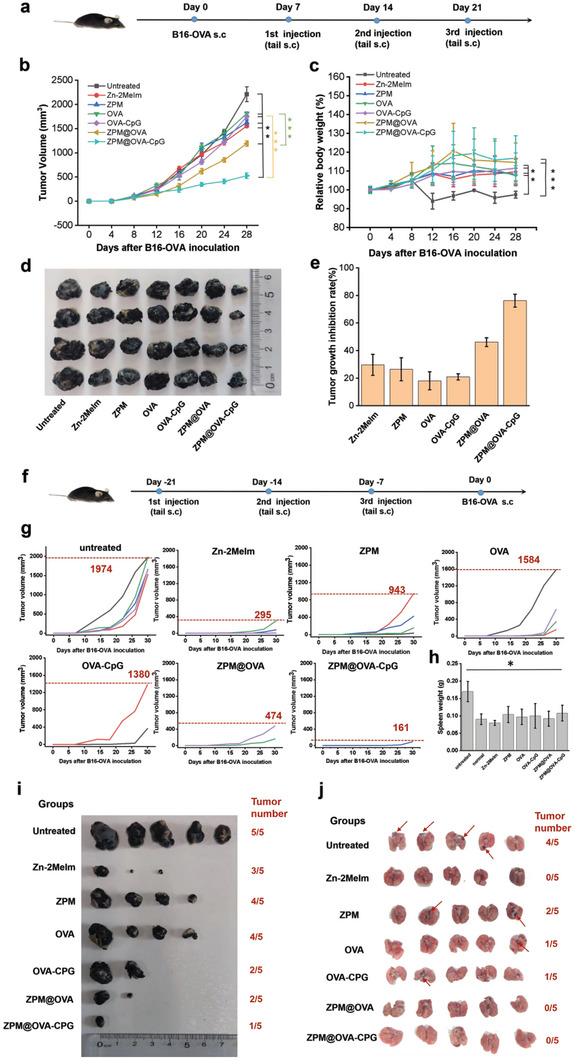
The efficacy verified of ZPM@OVA‐CpG vaccine by animal models. a‐e) Therapeutic studies, (a) therapeutic process; (b) tumor volume growth curves, data presented as mean ± SD (*n* = 4); (c) weight change curves, data presented as mean ± SD (*n* = 4); (d) tumor photography; (e) tumor growth inhibitory rate, data presented as mean ± SD (*n* = 4). f‐i) Prevention studies, (f) prophylactic process; (g) tumor growth curves for each mouse; (h) spleen weight, data presented as mean ± SD (*n* = 5); (i) tumor formation was photographed in the prevention groups. j) Imagings of lung metastases. *p < 0.05; **p < 0.01; ***p < 0.001.

Melanoma mouse models were treated with each group, and the experimental process was shown in **Figure** **7**a. The results showed that the ZPM@OVA‐CpG group had the best inhibitory effect on melanoma growth (Figure 7b,d). The relative body weight of untreated mice was significantly lower than that of other groups (Figure 7c). On day 28, the tumor was removed and measured in volume. The tumor inhibition rate was calculated: (1‐ average tumor volume of the experimental group / average tumor volume of the no treatment group) × 100%. The tumor inhibitory rates of Zn‐2MeIm, Man‐Zn, OVA and OVA‐CpG groups were 29.7%, 26.4%, 18%, and 20.9%, respectively, indicating that the antitumor effect of Zn‐MOF carriars were better than that of antigen‐adjuvant group. The inhibitory rate in the ZPM@OVA group was 46.1%, which was 28.1% higher than that in the OVA group. The inhibitory rate of ZPM@OVA‐CpG was 76.2%, which was 55.3% higher than that in the OVA‐CpG group (Figure 7e). These results indicate that ZPM@OVA‐CpG vaccine effectively inhibited the growth of melanoma, and the therapeutic effect is significantly better than that of single injection of antigen and antigen‐adjuvant groups. Moreover, after 28 days of treatment, the tumor average volume of 525 mm^3^ in the ZPM@OVA‐CpG vaccine group was less than that of other reported nano vaccines, indicating the advantages of ZPM@OVA‐CpG vaccine in treating solid tumors.^[^ ^55^, ^56^, ^57^ ^]^

Subsequently, we explored whether the experimental group could effectively prevent tumor formation (Figure 7f). The tumor growth curve showed that no tumor was observed in the ZPM@OVA‐CpG group within 20 days after subcutaneous injection of tumor cells, delayed tumor formation for the longest time (Figure 7g). The largest tumors (161 mm^3^) in the ZPM@OVA‐CpG group were the smallest relative to the largest tumors in the other groups (Figure 7g,i). Inhibition of tumorigenesis rates from high to low is ZPM@OVA‐CpG (80%), ZPM@OVA (60%), OVA‐CpG (60%), Zn‐2MeIm (40%), ZPM (20%) and OVA (20%) (**Table** **1**). And we found that the weight of the spleen in the untreated group was significantly higher than that in the treatment groups, showing the pathological changes in the spleens of tumor mice (Figure 7h). These results indicated that the ZPM@OVA‐CpG vaccine group had a high inhibitory effect on tumorigenesis.

**Table 1:** Tumor‐free proportion statistics (%)

	untreated	Zn‐2MeIm	ZPM	OVA	OVA‐CpG	ZPM@OVA	ZPM@OVA‐CpG
Prevention group	0	40	20	20	60	60	80
Metastasis group	20	100	60	80	80	100	100

Tumor metastasis in vivo is an important cause of death in cancer patients. Therefore, we also studied the killing effect of the experimental group on metastatic cancer cells. We injected B16‐OVA tumor cells into mice through the tail vein, and injected each experimental group into mice 7 days later (three times in total, with an interval of 7 days). 7 days after the three times of injection, we dissected the mice, took out the lungs, and observed the growth of pulmonary metastatic tumors. As shown in Figure 7j, black metastatic tumor tissue (shown by the red arrows) appeared in the lungs of four mice in the untreated group. No metastasis was observed in the Zn‐2MeIm, ZPM@OVA, and ZPM@OVA‐CpG groups, the inhibition efficiency reached 100%, while OVA and OVA‐CpG groups inhibition efficiency were 80% (Table 1).

In conclusion, according to the above animal experiments, the ZPM@OVA‐CpG vaccine not only showed the most lethal ability against solid tumors, but also effectively inhibited the formation and metastasis of tumors, greatly improving the anti‐tumor effect of the vaccine.

## 3 Conclusion

In summary, we designed a novel Zn‐MOF vaccine, which released Zn^2+^ in the DCs lysosome and tumor microenvironment. The Zn‐MOF vaccine strongly amplified the cGAS‐STING signal, stimulated DCs maturation and antigen cross‐presentation, and then activated the activation and proliferation of CD8^+^ T cells, inducing a strong specific immune response. In addition, the vaccine regulated MMP‐2 activity and degraded various collagen components of tumor ECMs, which broke the ECM barrier, alleviated the immunosuppressive environment, and enhanced CD8^+^ T cells tumor invasion and killing. The Zn‐MOF vaccine not only solved the bottleneck of vaccine efficacy, but also revealed the important role of metal ions in mediated immune activation and regulation of tumor microenvironment, which provided a new idea for the in‐depth intersection and development of nanoscience, materials science, and tumor immunology.

## 4 Experimental Section

### Materials, Cell Lines, and Mice

Zn(NO_3_)_2_·6H_2_O (98%), 2MeIm (98%), PEI (N.W. 600, 99%), Man (99%), MnCl_2_, ZnCl_2,_ CuCl_2_, KCl, FeCl_2_, CaCl_2_, MgCl_2_, were purchased from Aladdin. OVA was obtained from Sigma‐Aldrich. FITC‐OVA was purchased from Xi 'an Haoran Biological Biotechnology Co., LTD. CpG oligodeoxynucleotides was obtained from Guangzhou A ji Biotechnology Co., LTD. CCK‐8 assay kit, lactate dehydrogenase assay kit, BCA protein assay kit, Lyso‐Tracker Red, DCFH‐DA fluorescent probe were purchased from Beyotime. ELISA kits were obtained from mlbio, Shanghai. Blood biochemical index kits were obtained from Elabscience, China. AIM‐V medium, X‐VIVO medium, DMEM medium were obtained from Termo Scientifc. Recombinant human GM‐CSF growth factor, recombinant human IL‐4 growth factor, recombinant human IL‐2 growth factor, recombinant human TNF‐*α* growth factor were purchased from PeproTech. T‐Select H‐2Kb OVA Tetramer‐SIINFEKL‐PE was obtained from MBL. EasySep Human CD8 Pos Sel Kit II, EasySep Mouse CD8a Positive Slctn Kit II, CFSE dye were obtained from Stemcell. Antibodies used in this study are listed in Table S3, Supporting Information.

PBMC was obtained from the Cell Bank of Shenzhen People's Hospital. B16‐OVA cells line were obtained from Fuheng Biology, Shanghai.

All the animals were cared for and treated according to the instructions and approval of the Institutional Animal Care and Use Committee of Shenzhen People's Hospital (LL‐KY‐2021603). Female C57BL/6 mice (5–6 weeks old) were purchased from Guangdong Yaokang Biotechnology Co. LTD.

### DCs / T Cells were Isolated from PBMC and Cultured in Vitro

PBMC were resuscitated from liquid nitrogen, the cells concentration were adjusted to 5 × 10^6^ cells per well by AIM‐V medium, and cultured in a 12‐well plate. After standing for 2 h in an incubator at 37 °C, the cells were shaken to remove the nonadherent cells in the upper layer, leaving the adherent cells, 800 µL AIM‐V medium included 200 ng mL^−1^ GM‐CSF and 100 ng mL^−1^ IL‐4 were added. After 3 days cultured, half of the solution was changed and cytokines were supplemented. On day 5, 400 µL AIM‐V medium included 200 ng mL^−1^ TNF‐a were added. On day 7, DCs were collected.

PBMC were resuscitated from liquid nitrogen, the cells concentration were adjusted to 5 × 10^6^ cells per well by AIM‐V medium, and cultured in a 12‐well plate. After standing for 2 h in an incubator at 37 °C, the upper layer of flocculent T cells were collected by shaking the cells. T cells were cultured in 24‐well plate with 800 µL of X‐VIVO medium included 20 ng mL^−1^ IL‐2.

Zn^2+^ stimulates cGAS‐STING pathway activation, up‐regulates MMP‐2 activity, and induces ROS production in tumor:

DCs were isolated from PBMC by the above method. DCs were cultured in 24‐well plate with the concentration of 1 × 10^6^ cells/well. MnCl_2_, ZnCl_2_, CuCl_2_, KCl, FeCl_2_, CaCl_2,_ and MgCl_2_ (250, 500, 1000 µM) were co‐cultured with DCs for 24 h, after centrifugation, supernatant was collected and IFN‐*α* and IFN‐*β* content were detected by ELISA kits.

l929 cells were cultured in 6‐well plate at the concentration of 2 × 10^5^ cells per well, and l929 cells were coincubated with 1000 µM ZnCl_2_ for 24 h. Then the cells were collected and the enzyme activity was detected using MMP‐2/9 gelatin enzyme profile kit. The methods were as follows: 1) The cells lysed with hypoosmotic buffer solution were centrifuged to collect proteins, and then subjected to routine electrophoresis until bromophenol blue was completely gelated. The gel was washed in an eluent of 2.5% Triton X‐100, 50 mM Tris‐HCl, 5 mM CaCl_2_, 1 µM ZnCl_2,_ and pH 7.6 twice for 40 min each time. 2) Rinse with bleach (50 mM Tris‐HCl, 5 mM CaCl_2_, 1 µM ZnCl_2_) twice for 20 min each time. 3) The gel was incubated in 37 °C medium (50 mM Tris, pH 7.5, 150 mM NaCl, 10 mM CaCl_2_, 1 µM ZnCl2, 0.02% Brij‐35) for 48 h. 4. Soak the Coomassie bright blue dyeing solution for 3 h, and finally decolorize the solution. Use the gel imager for imaging.

B16‐OVA cells were cultured in 6‐well plate at the concentration of 2 × 10^5^ cells per well for overnight incubation, then was cocultured with 1000 µM ZnCl_2_ for 6 h. DCFH‐DA fluorescent probe was used to label ROS generated in tumor cells for imaging observation using fluorescence microscopy.

DCs, T cells (1 × 10^4^ cells per well), and B16‐OVA cells (1 × 10^4^ cells per well) were cultured in 96‐well plate. ZnCl_2_ (0, 250, 500, 1000, 1500 µM) were cocultured with cells for 24 h and then cytotoxicity were then measured using CCK‐8 kits.

Preparation and characterization of Zn‐2MeIm, ZPM and ZPM@OVA‐CpG:

To prepare Zn‐2MeIm, Zn(NO_3_)_2_·6H_2_O (3 mL, 0.05 g) and 2MeIm (2 mL, 0.97 g) dissolved in water were mixed together followed by stirring at 500 rpm for 15 min at room temperature. The products were collected by centrifugation (10 000 rpm for 15 min), washed twice with water, and then dried under vacuum at 40 °C.

For the synthesis of ZPM, 0.05 g Zn(NO_3_)_2_·6H_2_O and 0.05 g PEI mixed dissolved in 3 mL deionized water to form solution 1, 0.97 g 2MeIm was dissolved in 2 mL deionized water to form solution 2. Solution 1 and solution 2 were mixed together followed by stirring at 500 rpm for 15 min at room temperature. The products were collected by centrifugation (10,000 rpm for 15 min). Subsequently, 3 mL of water was added to the product and dispersed by sonication to form solution 3. 0.5 g Man was dissolved in 2 mL deionized water to form solution 4. Solution 3 and solution 4 were mixed together followed by stirring at 500 rpm for 30 min at room temperature. The products were collected by centrifugation (10,000 rpm for 15 min), washed twice with water, and then dried under vacuum at 40 °C.

To prepare ZPM@OVA‐CpG, 0.05 g Zn(NO_3_)_2_·6H_2_O and 0.05 g PEI mixed dissolved in 2.5 mL deionized water to form solution 1, 0.97 g 2MeIm, 0.005 g OVA and 150 µg CpG‐ODN were dissolved in 2.5 mL deionized water to form solution 2. Solution 1 and solution 2 were mixed together followed by stirring at 500 rpm for 15 min at room temperature. The products were collected by centrifugation (10,000 rpm for 15 min). Subsequently, 3 mL of water was added to the product and dispersed by sonication to form solution 3. 0.5 g Man was dissolved in 2 mL deionized water to form solution 4. Solution 3 and solution 4 were mixed together followed by stirring at 500 rpm for 30 min at room temperature. The products were collected by centrifugation (10,000 rpm for 15 min), washed twice with water, and then dried under vacuum at 40 °C. To explore the loading capacity of ZPM for OVA, different concentrations of OVA (0.3, 0.5, 1, 1.5, 2, 2.5 mg mL^−1^) were set during the preparation of ZPM@OVA‐CpG. After the reaction, the supernatant was collected by centrifugation, and the free OVA content of the supernatant was detected by BCA Kit. It was calculated by the formula: encapsulation rate % = (total amount of OVA‐ supernatant amount of OVA)/total amount of OVA×100%.

To investigate the OVA release efficiency of ZPM in acidic environment, ZPM@OVA‐Cpg (2 mg mL^−1^) were dispersed in PBS (with pH of 7.4, 6.5, or 5) at 37 °C under stirring. At desired time intervals (0.25 h, 0.5 h, 1 h, 2 h, 4 h, 8 h, 16 h, 24 h), samples were centrifuged (10 000 rpm for 30 min) and the supernatant was collected for the measurement of OVA amount by BCA assay. Release rate % = supernatant amount of OVA/total amount of OVA×100%.

ZPM and ZPM@OVA‐CpG powder (1 g) were dispersed in PBS solution to observe its dispersivity. The stability was investigated by measuring the change of UV absorption peaks in pH 7.4 for 7 days. ZPM and ZPM@OVA‐CpG (0.5 g) were dispersed in PBS solution with different pH (7.4, 6.5, 5), photographs were taken at 0 h and 6 h to observe whether the solution became clear, and the released Zn^2+^ were measured with a Zn colorimetric kit. Morphology changes of ZPM@OVA‐CpG were observed by SEM.

The morphology of products were observed by TEM (JEM‐2100F, JEOL, Japan) and SEM (SEM Prisma E, Thermo Scientific, USA). The size distribution and zeta potential of the products were analyzed using a Nano Zetasizer (Malvern, Worcestershire, UK). FTIR analyses were carried out with Nicolet iS50 Spectrometer (Thermo Scientific, USA). PXRD were detected by D2 PHASER X‐ray powder (Bruker, Germany). The UV absorption peaks of the products were characterized by LAMBDA 950 (PerkinElmer, USA)

### Biosafety Analysis

Histopathological observation, blood biochemistry, organ index, and weight recorded:

Female C57BL/6 mice (5–6 weeks old) were subcutaneously injected with different groups (at an equivalent OVA amount of 50 µg per mice) for 3 times with an interval of 7 days. On day 21, the weight of the mice was weighed, and the blood of the mice were collected by taking blood from the orbit. The blood was left at room temperature for 2 h, followed by 4 °C overnight. Next day, the samples were centrifuged at 3000 rpm for 10 min, and the supernatant was collected and detected with a blood biochemical index kit. The mice were dissected, and the organs were collected and weighed to calculate the organ index. organ index = organ weight (g)/body weight (g) Pathological sections were prepared and observed after HE staining.

### Cytotoxicity to DCs and T Cells

DCs, T cells (1 × 10^4^ cells per well) and B16‐OVA cells (1×10^4^ cells/well) were cultured in 96‐well plate. Zn‐2MeIm, ZPM and ZPM@OVA‐CpG (0, 25, 50, 100 mg L^−1^) were co‐cultured with cells for 24 h and then cytotoxicity were then measured using CCK‐8 kits.

ZPM@OVA‐CpG vaccine target DCs to promote antigen uptake and presentation

### In vivo lymph node targeting

FITC‐OVA‐CpG and FITC‐ZPM@OVA‐CpG (at an equivalent OVA amount of 50 µg per mice) were subcutaneously injected into mice, the distribution and lymph node targeting of the antigen in vivo were observed and recorded by in vivo imaging system at 0.5 h, 6 h, 12 h, and 24 h. Fluorescence intensity values were recorded. 24 h after injection, the inguinal lymph nodes of mice were removed and their fluorescence signals were observed.

### DCs Uptake Antigen and Lysosome Sscape

DCs were isolated from PBMC, cells (1 × 10^6^ cells per well, 1 mL) were seeded into confocal dishes, on day 5 of culture, FITC‐OVA‐CpG and FITC‐ZPM@OVA‐CpG (at an equivalent OVA concentration of 50 mg L^−1^ were addeed. After 6 h incubation, cell fluorescence signals were observed and recorded by confocal microscopy, lysosomes were labeled with LysoTracker Red.

### The cGAS‐STING Pathway is Activated to Release IFN‐*α* and IFN‐*β*

DCs were isolated from PBMC, cells (1 × 10^6^ cells per well, 1 mL) were seeded into 24‐well plate. On day 5 of cultured, DCs were cocultured with each experimental group (50 mg L^‐1^) for 24 h, and then centrifuged the plates to collect supernatant. ELISA kits were used to detect the contents of IFN‐*α* and IFN‐*β* released in the culture solution. EDTA, as Zn^2+^ chelator, was mixed with ZPM@OVA‐CpG group (EDTA : Zn^2+^ molar ratio 1:1) to become ZPM@OVA‐CpG + chelator group.

### DC Maturity

DCs were isolated from PBMC, cells were seeded into 24‐well plate (1 × 10^6^ cells per well). On day 5 of culture, the different experimental groups were co‐cultured with DC for 24 h. Subsequently, DC were collected and incubated with CD16/32 antibody at room temperature, and then stained with fluorescently labeled FITC‐anti‐human CD11c, (B7‐2) PE‐Cy7‐anti‐human CD86 and APC‐anti‐human CD83 antibodies for 30 min and subsequently detected by flow cytometry.

### Antigen Cross‐Presentation

DCs were isolated from PBMC, cells were seeded into 24‐well plate (1 × 10^6^ cells per well). On day 5 of cultured, the different experimental groups were co‐cultured with DCs for 24 h. Subsequently, DCs were collected and incubated with CD16/32 antibody at room temperature, and then stained with fluorescent labeled FITC‐anti‐human CD11c and APC‐anti‐human HLA‐A2 antibodies for 30 min and subsequently detected by flow cytometry.

CD8^+^ T cells proliferation activation and tumor‐killing

### T Cell Proliferation

DCs were seeded into 24‐well plate (1 × 10^6^ cells per well, 1 mL), on day 5 of cultured, each experimental group (50 mg L^−1^) were added for cocultured of 24 h in advance, and then treated DCs were collected and cocultured with CFSE‐labeled T cells for 3 days. CFSE fluorescence was analyzed by flow cytometry to detect T cell proliferation.

### Total T Cells and CD4^+^/CD8^+^ T Cells Ratio in Spleen

To investigate whether the experimental groups could effectively increase the total number of T cells and the proliferation of CD8^+^ T cells in vivo, each experimental group was injected subcutaneously into C57 mice for a total of 3 times, each time at an interval of 7 days. On day 21, the spleen of mice was collected and sliced to prepare a single‐cell suspension, which was incubated with red cell lysate and centrifuged for filtration. The cells were treated with T‐Select H‐2Kb OVA Tetramer‐SIINFEKL‐PE to sort out OVA‐specific T cells. The tetramerers were incubated and stained with APC‐anti‐mouse CD8a, FITC‐anti‐mouse CD3, and PE‐Cy7‐anti‐mouse CD4 antibodies. Total OVA‐specific T cells count and CD4^+^/CD8^+^ T cells ratio were measured by flow cytometry.

### Detection of Cytokines Secreted by CD8^+^ T Cells

DCs were isolated from PBMC and inoculated into 24‐well plates (1 × 10^6^ cells per well). On day 5 of cultured, different groups (50 mg L^−1^) were cocultured with DCs for 12 h, then 1 × 10^7^ sorted CD8^+^ T cells were added to each well for 48 h, centrifuged and supernatant was collected, and the content was detected by IFN‐*γ*, TNF‐*α*, and IL‐4 ELISA kits, respectively.

### The Ability of Activated CD8^+^ T Cells to Kill Cancer Cells

DCs were isolated from PBMC and inoculated into 24‐well plates (1 × 10^6^ cells per well). On day 5 of cultured, OVA‐CpG and ZPM@OVA‐CpG groups (50 mg L^−1^) were cocultured with DCs for 12 h, then 1 × 10^7^ sorted CD8^+^ T cells were added to each well for 3 days. The T cells were centrifugally washed and collected. Then with B16‐OVA cells as target cells and the above‐induced T cells as effector cells, cells were cultured in 96‐well plate at the effect‐target ratio of 5:1, 10:1, and 15:1. After co‐cultured for 24 h, the apoptosis rate of cancer cells was detected by lactate dehydrogenase kit.

ZPM@OVA‐CpG vaccine degrades tumor ECM and relieves the immunosuppressive environment

### Transcriptome Sample Preparation and Analysis

B16‐OVA cells were grown in petri dishes (1 × 10^6^ cells/dish), after overnight incubation, ZPM@OVA‐CpG (50 mg L^−1^) were added and cocultured with B16‐OVA cells for 24 h, and then collected the cells and washed with TRIzol reagent until no cells were visible. The solution was in a clear state, stored at −80 °C, and sent to Novogene for transcriptomic detection. Untreated B16‐OVA cells served as controls. Data analysis was conducted on the cloud platform of Novogene.

### Detection of MMP Enzyme Activity

l929 cells were cultured in 6‐well plate (2 × 10^5^ cells per dish) for overnight, and then l929 cells were coincubated with ZPM@OVA‐CpG (50 mg L^−1^) for 24 h. Then the cells were collected and the enzyme activity was detected using MMP‐2/9 gelatin enzyme profile kit.

### Western Blot Analysis

B16‐OVA cells were grown in petri dishes (1 × 10^6^ cells per dish), after overnight incubation, B16‐OVA cells were cocultured with ZPM@OVA‐CpG vaccine group, OVA‐CpG and ZPM@OVA‐CpG + chelator groups (50 mg L^−1^) for 24 h, then the cells were ice‐dissolved in RIPA lysis buffer containing protease inhibitor for 20 min, supernatant was collected, and protein quantification was performed by BCA method.

Take 30 µg of protein and added it into the sample buffer, denatured at 100 °C for 10 min, SDS‐PAGE was prepared, samples were taken, 120 V electrophoresis was performed for 60 min, the protein was transferred to PVDF membrane, and 5% skim milk powder was closed for 60 min. GAPDH, Ltbp4, Lamb2, Col20a1, Col15a1, Col6a3, and Itgb5 antibodies were given in 1:1000 dilution. Incubated overnight at 54 °C, washed the film with PBST 3 times, 10 min each time, added horseradish peroxidase‐labeled IgG, incubated for 1 h. PBST film washing 3 times, 10 min each time, ECL color, dark room exposure, scanning, using ImaseJ software to get gray value, to control the area gray value of 100% and the experimental group for comparison and semi‐quantitative analysis.

### IL‐10 and TGF‐*β* Detection

B16‐OVA cells were cultured in 6‐well plate (2 × 10^5^ cells per dish) for overnight, and then cells were coincubated with ZPM@OVA‐CpG (50 mg L^−1^) for 24 h, then centrifuged, and the supernatant was collected, the IL‐10 and TGF‐*β* contents were detected by ELISA kits, respectively.

### ROS Generation Detection

B16‐OVA cells were cultured in 6‐well plates (2 × 10^5^ cells per dish) overnight, and then cells were co‐incubated with ZPM@OVA‐CpG (50 mg L^−1^) for 6 h. DCFH‐DA fluorescent probe was used to label ROS generated in tumor cells for imaging observation using fluorescence microscopy.

ZPM@OVA‐CpG vaccine inhibit tumor growth, prevent tumor formation and metastasis

### Therapeutic Studies

For the therapeutic tumour studies, C57BL/6 mice were inoculated with 1 × 10^6^ B16‐OVA cells per mouse on the right flank by subcutaneous injection. 7 days after the injection of tumor cells, mice were injected three times with different experimental groups (at an equivalent OVA amount of 50 µg per mice) at an interval of 7 days, the tumor size, and weight of mice were monitored every four days. Tumor volume: *V* = length × width × width/2 (mm^3^). On day 28, the mice were killed, the tumor was removed, weighed, photographed, and the tumor inhibition rate was calculated.

### Prevention Studies

To prevent tumor formation studies, mice were injected three times with different experimental groups at an interval of 7 days (at an equivalent OVA amount of 50 µg per mice). On day 21, 1 × 10^5^ B16‐OVA cells were injected subcutaneously into the upper right thigh of mice. The tumor growth of mice was monitored every 5 days, and the mice were killed on the 30th day. The tumors were removed, photographs, tumor growth curves were drawn, and the tumor‐free proportion was calculated.

### Metastasis Study

To study the effect of inhibiting tumor metastasis, mice were injected with 1 × 10^5^ B16‐OVA cells through the tail vein. 7 days after injection of tumor cells, mice were injected three times with different experimental groups at an interval of 7 days (at an equivalent OVA amount of 50 µg per mice). 7 days after the completion of 3 times of injection, the mice were sacrificed and lung tissue was removed to observe the metastatic growth of lung tumors.

## Conflict of Interest

The authors declare no conflict of interest.

## Supporting information

Supporting Information

## Data Availability

The data that support the findings of this study are available from the corresponding author upon reasonable request.
