# Supplementary material for: Zinc‐Organometallic Framework Vaccine Controlled‐Release Zn2+ Regulates Tumor Extracellular Matrix Degradation Potentiate Efficacy of Immunotherapy
Source: Adv Sci (Weinh). 2023 Jul 13;10(27):2302967. doi: 10.1002/advs.202302967 (PMC10520680; doi:10.1002/advs.202302967)
Supplement: Supplementary file 1 — Supporting Information [file ADVS-10-2302967-s001.pdf]

ADVANCED SCIENCE
Open Access

Supporting Information

for *Adv. Sci.*, DOI 10.1002/advs.202302967

Zinc-Organometallic Framework Vaccine Controlled-Release Zn^{2+} Regulates Tumor Extracellular Matrix Degradation Potentiate Efficacy of Immunotherapy

Lin Ding, Minli Liang, Yuanyuan Li, Mei Zeng, Meiting Liu, Wei Ma, Fuming Chen, Chenchen Li, Rui L. Reis, Fu-Rong Li and Yanli Wang**

Supporting Information

Zinc-organometallic framework vaccine controlled-release Zn^{2+} regulates tumor extracellular matrix degradation potentiate efficacy of immunotherapy

Lin Ding,[1,3,4] Minli Liang,[1,3,4] Yuanyuan Li,[5] Mei Zeng,[6] Meiting Liu,[6] Wei Ma,[3] Fuming Chen,[3] Chenchen Li,[2] Rui L. Reis,[7] Fu-Rong Li,[1,3,4] Yanli Wang,[2]**

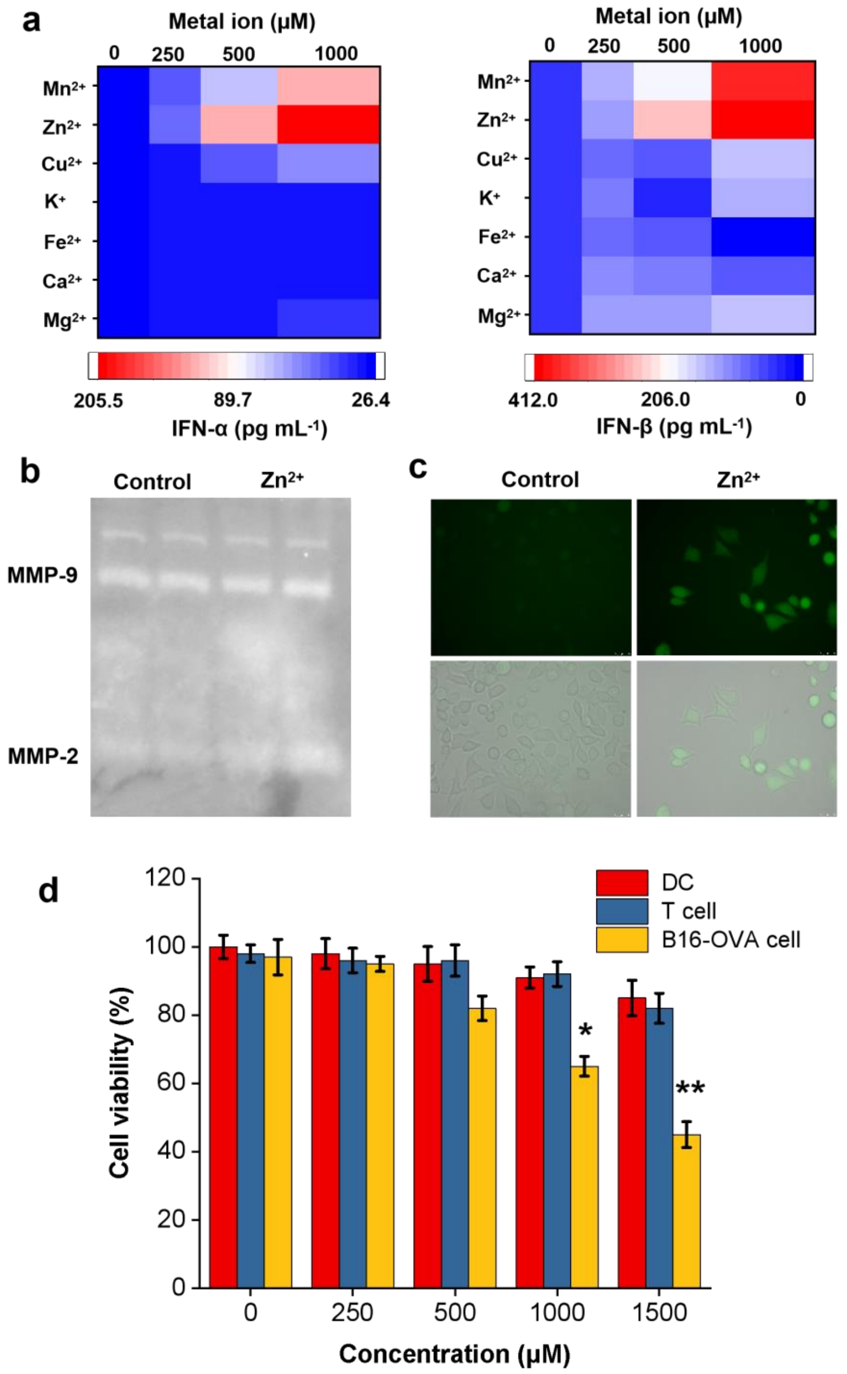

Figure SI 1. Zn^{2+} stimulates cGAS-STING pathway activation, up-regulates MMP-2 activity, and induces ROS production in tumor. (a) The contents of IFN-α and IFN-β secreted by DCs were detected; (b) enzyme activity bands of MMP-2/9; (c) intracellular ROS generated fluorescent labeling imaging; (d) cytotoxicity of Zn^{2+} on DCs, T cells and B16-OVA tumor cells, data presented as mean ± SD (n=6).

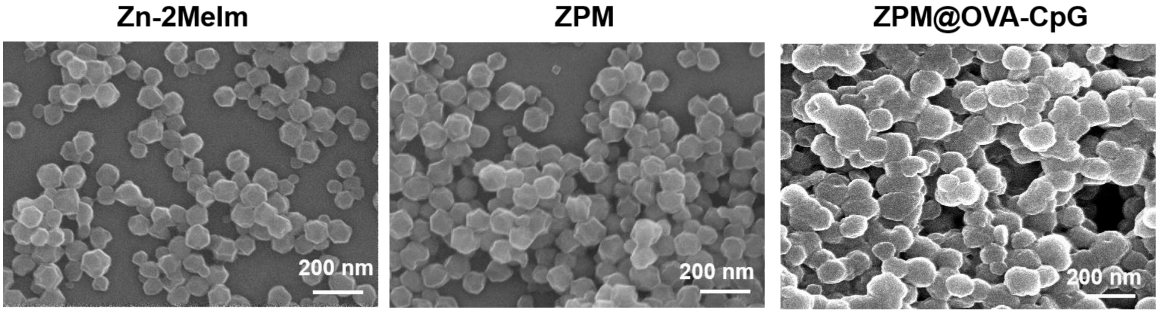

Figure SI 2. SEM images of Zn-2MeIm carrier, ZPM carrier and ZPM@OVA-CpG.

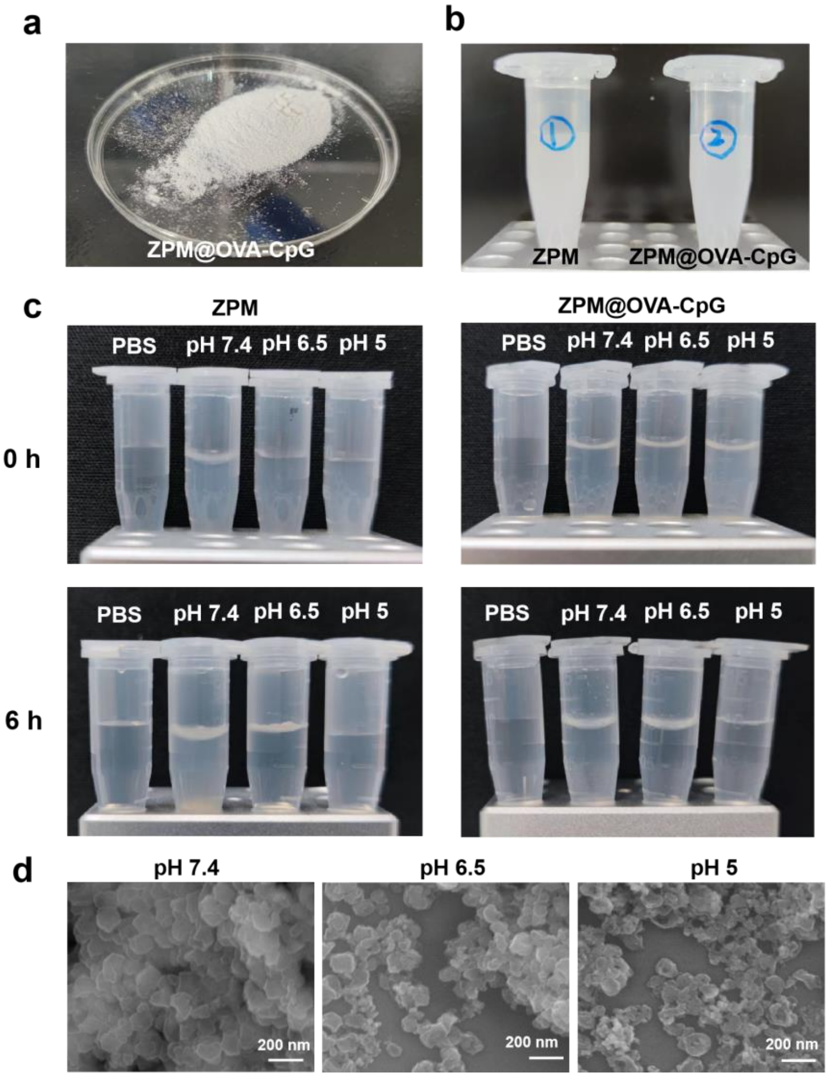

Figure SI 3. (a) ZPM@OVA-CpG appears as white powder; (b) ZPM and ZPM@OVA-CpG were uniformly dispersed in PBS; (c) ZPM and ZPM@OVA-CpG were cleaved at different pH of 0 h and 6 h; (d) morphology changes of ZPM@OVA-CpG after different pH treatment for 6 h.

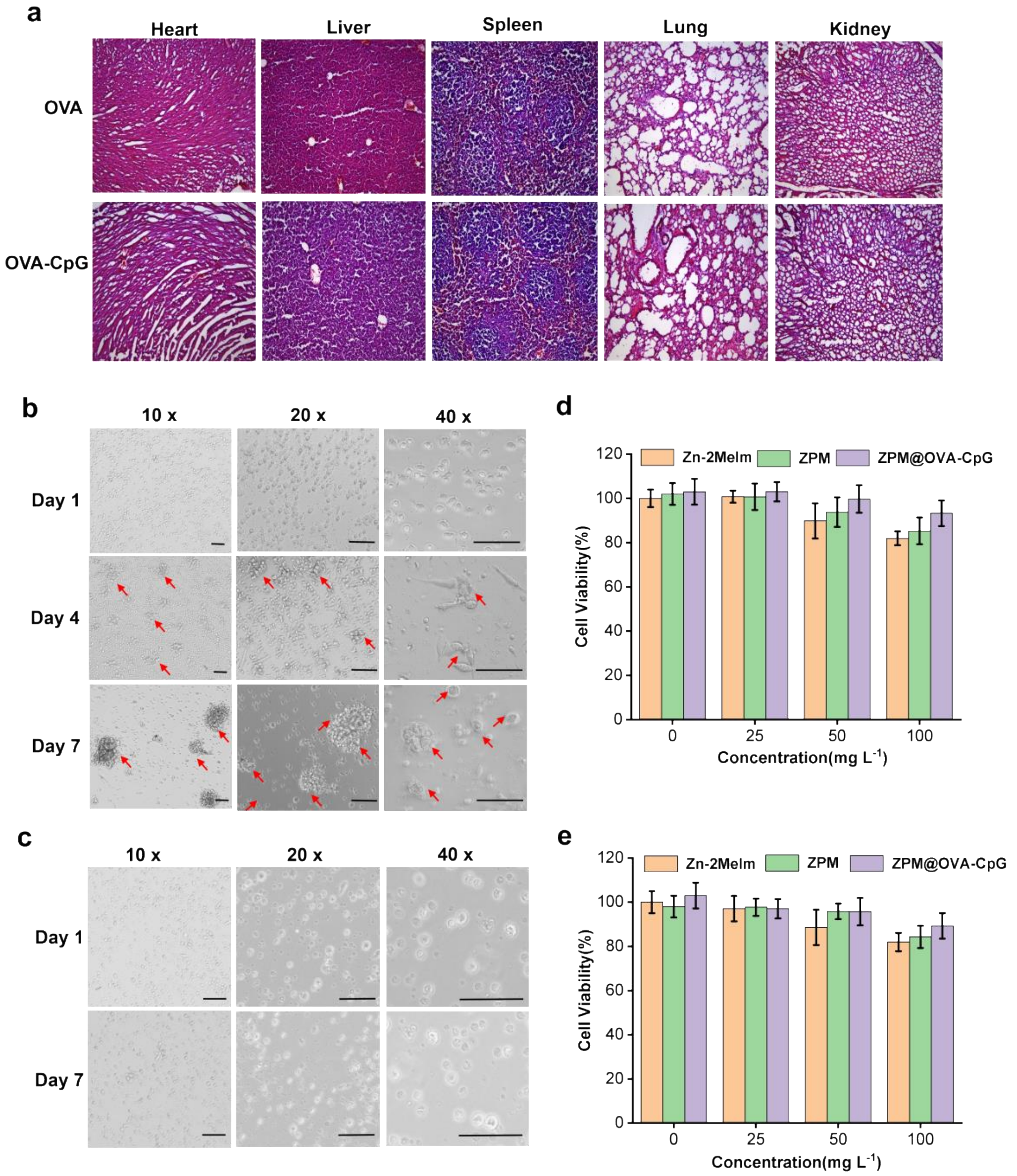

Figure SI 4. (a) Pathological section observation of OVA and OVA-CpG groups; (b) DCs induced culture in vitro; (c) T cells induced culture in vitro; (d) cytotoxicity of Zn-2MeIm, ZPM and ZPM@OVA-CpG to DCs, data presented as mean ± SD (n=6); (e) cytotoxicity of Zn-2MeIm, ZPM and ZPM@OVA-CpG to T cells, data presented as mean ± SD (n=6). Scale is 50 μm.

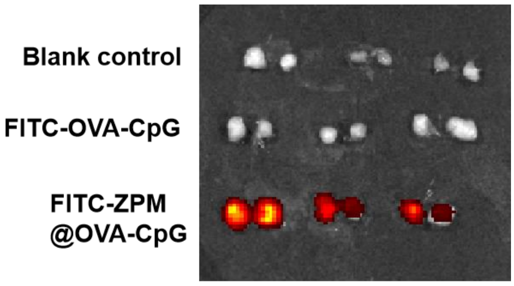

Figure SI 5. Fluorescence imaging of ex-lymph nodes.

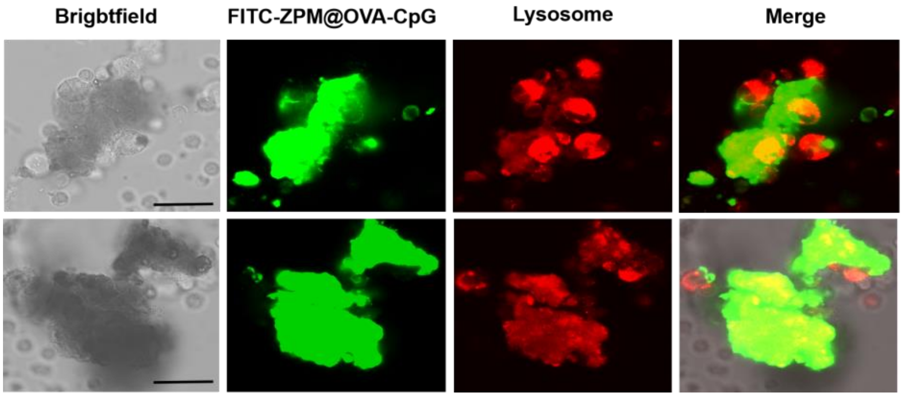

Figure SI 6. Confocal imagings of FITC-ZPM@OVA-CpG uptake by DC clusters. Scale is 50 µm.

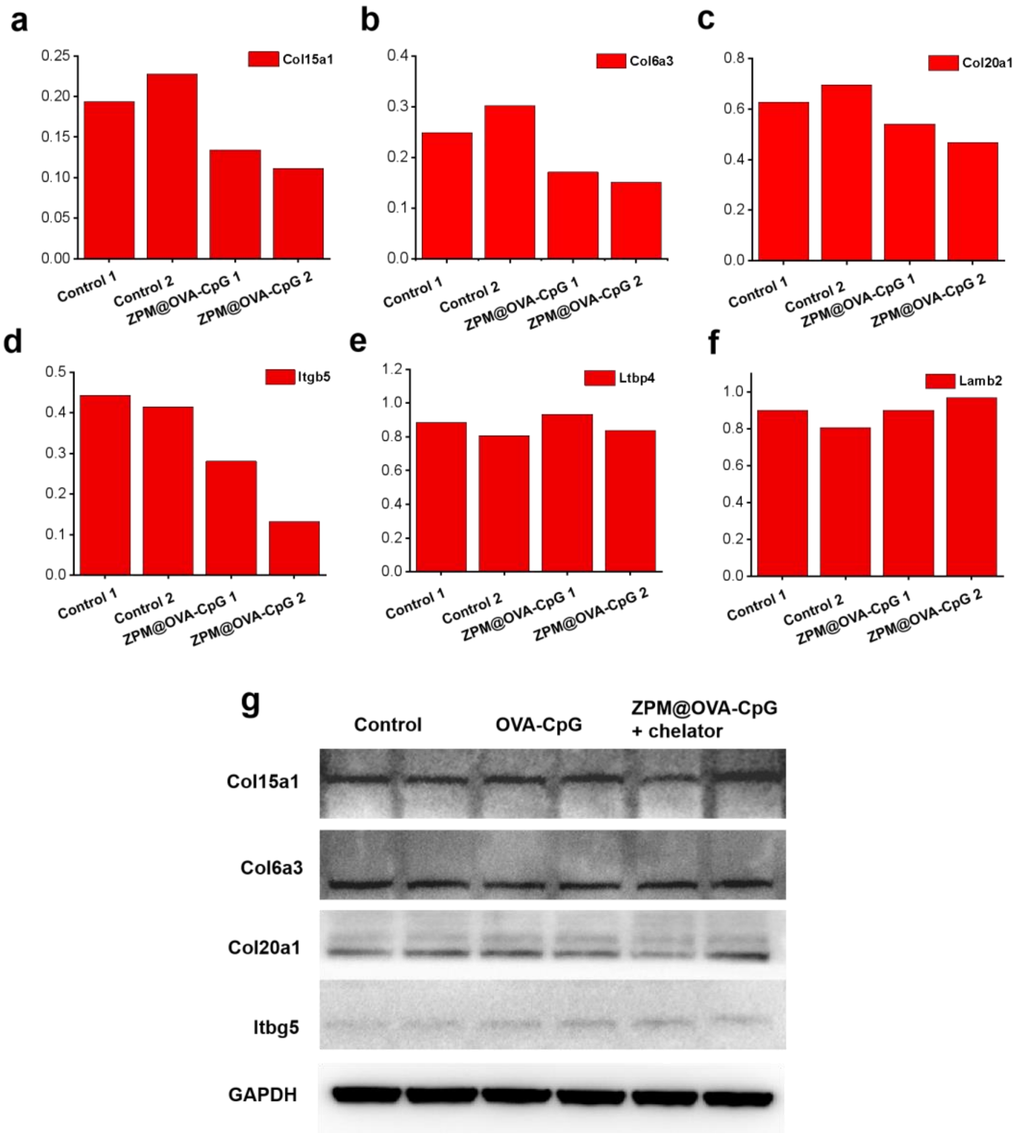

Figure SI 7. Column charts of average integrated density val/ GAPDH average integrated density val of western blotting bands (ratio) . (a) Col15a1; (b) Col16a3; (c) Col20a1; (d) Itgb5; (e) Ltbp4; (f) Lamb2; (g) western blot verification.

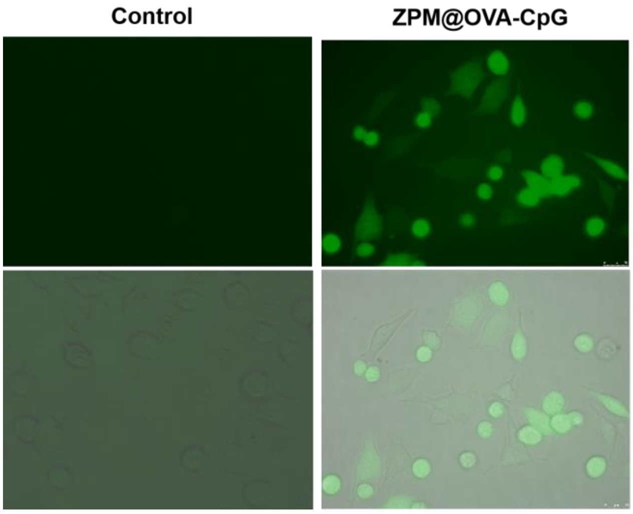

Figure SI 8. ZPM@OVA-CpG induced ROS production in tumor cells.

Table SI 1. Average integrated density val of western blotting bands

	Col15a1	Col6a3	Col20A1	Itgb5	Ltbp4	Lamb2	GAPDH
Control 1	47.92	61.57	154.638	109.448	218.703	221.998	247.027
Control 2	56.237	74.517	171.688	102.267	199.646	198.728	248.456
ZPM@OVA-CpG 1	33.184	42.239	133.589	69.369	230.791	222.202	253.267
ZPM@OVA-CpG 2	27.581	37.453	115.176	32.688	206.79	239.119	252.347

Table SI 2. Average integrated density val/ GAPDH Average integrated density val (ratio)

	Col15a1	Col6a3	Col20A1	Itgb5	Ltbp4	Lamb2	GAPDH
Control 1	0.1939	0.2492	0.6259	0.4430	0.8853	0.8986	0.1939
Control 2	0.2276	0.3016	0.6950	0.4139	0.8081	0.8044	0.2276

ZPM@OVA-CpG 1	0.1343	0.1709	0.5407	0.2808	0.9342	0.8995	0.1343
ZPM@OVA-CpG 2	0.1116	0.1516	0.4662	0.1323	0.8371	0.9679	0.1116

Table SI 3. Antibody list

Antibody	Manufacturer	Product number	Dilutions
Anti-Human CD83 APC	PeproTech	05911-80	1:200
Anti-Human CD86（B7-2） PE-Cy7	PeproTech	08911-77	1:200
FITC anti-mouse CD3 (17A2)	TONBO	35-0032-U100	1:200
PE/Cyanine7 anti-mouse CD4 Antibody	Biolegend	100422	1:200
APC anti-mouse CD8a Recombinant Antibody	Biolegend	155006	1:200
APC anti-mouse H-2Kb bound to SIINFEKL Antibody	Biolegend	141605	1:200
FITC anti-human CD11c Antibody	Biolegend	337214	1:200
APC anti-human HLA-A2 Antibody	Biolegend	343307	1:200
RabbitAnti-Integrin beta 5 antibody	Bioss	bs-23987R	1:1000
RabbitAnti-LTBP4 antibody	Bioss	bs-5779R	1:1000
RabbitAnti-COL15A1 antibody	Bioss	bs-0547R	1:1000
RabbitAnti-COL20A1 antibody	Bioss	bs-5881R	1:1000
RabbitAnti-COL6A3 antibody	Bioss	bs-0553R	1:1000
RabbitAnti-LTBP4 antibody	Bioss	bs-5779R	1:1000
